# Belladonna in the Treatment of Typhoid Fever

**Published:** 1870-08

**Authors:** B. Kelly

**Affiliations:** Dublin


### Miscellaneous.

### Belladonna in the Treatment of Typhoid Fever.

By Dr. B. Kelly, Dublin.

Without entering into the fruitless question as to whether fevers be due to specific germs, or to pythogenic fluids or gases, all of which, up to the present, have failed to be detected by the microscope and the most searching chemical tests, I would merely state that my own convictions lean strongly to the theory that this class of diseases owe their origin to the introduction into the circulation of septic vapors or gases, and that these subtle agents (fomites) are not unfrequently developed on the surface of the bodies of the individuals subsequently tainted by them. Hence the prevalence of these maladies among the poor, and all those who from ignorance or other circumstances ignore the practice of frequent ablutions and personal cleanliness. But more, there must exist, as a *sine quà non,* a predisposition on the part of the patient, as well as an exciting cause for the effectual production of fever. Now, of all the predisposing elements, there is none, perhaps, more powerful than poverty, with its concomitant train of filth, squalidness and misery.

The treatment of fever has been as conflicting and diversified as have been the opinions of physicians respecting its pathology. When the inflammatory theory was in vogue, bleeding, blistering and purging was pressed into service with terrible effect, as shown by the awful mortality which attended this form of treatment. And it has not seldom happened that the disease had been mistaken by a similarity in outward appearance with the phenomena of some local phlegmasia, and doctored accordingly, as we have good reason to suppose, judging from the lesions presented in *post mortem* examinations. No one can read the clinical works of Brousais without being seriously convinced of the truth of this statement. Now that the zymotic, or putrid and germinal theories reign supreme among the more orthodox members of the profession, the antiseptic plan of medication, based, as I believe it to be, upon sounder pathological doctrines, has superseded the more heroic method alluded to, and, comparing the results of both, with decided benefit to patients.

The treatment which I now propose has neitherbee n predicted by the inflammatory nor yet by the zymotic nature of the disease, and consequently cannot be styled antiphlogistic or antiseptic, at least in the ordinary acceptation of these terms; and yet, viewing it in the light of its action in preventing congestions and inflammations, and dissipating the putrid epiphenomena of fever, it well deserves the title of both.

Nearly six years have now elapsed since I recommended, on physiological grounds, belladonna in the treatment of enteric fever. During this time ample opportunity has been given me of testing its therapeutic virtues. Believing firmly, as I did, in the correctness also of the pathological deductions upon which the new treatment was especially based, I entered, I must confess, upon the trial with rather sanguine hopes of success. Never, I can say, have expectations been more fully realized or crowned with happier results. Indeed, the greatest difficulty I now find is to speak of it in such a way as not to detract from its merits by false moderation, or, by falling into the opposite and equally reprehensible extreme, to puff it up with unbounded praise. Suffice it to say that not more effectually is the tetanic action of strychnine on the system neutralised by tobacco and woorari, or paludal fever combated by quinine, or anæmia removed by chalybeates, than is the poison of typhoid counteracted by belladonna. It completely changes the whole character and outward manifestation of the disease. Delirium, coma and subsultus quickly vanish, and are succeeded by calmness and clearness of the intellect, by natural sleep, and complete control of all the voluntary muscles. Diarrhœa is checked, and healthy consistent evacuations are established. The appetite, if excessive or deficient, is restored to something like a normal standard. The pulse, from being frequent, fluttering and compressible, is rendered slow, strong and equable. The morbid temperature of the body—the *calor mordax* of Roman, the *causus* of Greek physicians—falls to a natural level. The vital changes induced in the blood, glandular and other organs, as shown by passive hemorrhages, tenderness of the abdomen, hypostatic congestions and ulcerations, are all arrested in *limine;* and the deranged functions of the economy return to their original equilibrium, and are performed with the same regularity as in health. The patient, after an inconceivably short space of time, usually from twenty-four to forty-eight hours, after the first administration of the remedy, wakes up, so to speak, and pronounces himself as well asever; and indeed, to look at him, he really appears so. If, however, the physician be not thoroughly on his guard, and do not keep the patient quiet in bed pending the ordinary course and duration of the disease, serious consequences will almost inevitably follow. This precaution is all the more necessary, as both the patient and his friends often become clamorous, and, unless gifted with more than a usual share of charity and subordination, may impute sinister motives to the ill-starred medical attendant. Under such circumstances he should never neglect to impress upon them the important fact that, favorable appearances to the contrary notwithstanding, the disease must run a eertain limited course; and that relapses, always dangerous in themselves, are only to be prevented by watching the patient closely, and combating untoward symptoms as they arise. These tactics I have never known to fail. Now a word as to the proper time and mode of administering belladonna.

When called to see a patient in fever, I usually wait, if it be yet in its incipient stage, till all or most of the prominent symptoms are well developed before I venture to prescribe the drug. By doing so, I have had the advantage and gratification of witnessing its effects in all their physiological force, while I avoid the possibility of making a mistake in diagnosis. If the patient be an adult, and vigorous, I do not hesitate to give from twenty to twenty-five drops of the officinal tincture of the British Pharmacopœia every four hours. This dose must necessarily vary to suit individual ages and constitutions. The following is the formula I generally employ:—℞. Tinct. belladonnæ ʒ ij., syrupi aurantii ℥ ss., aquæ menth pip. ℥ vijss. Ft. mist. Dosis: Pars sexta omni quarta hora sumenda. As the whole of this mixture is usually consumed in twenty-four hours, and as it is repeated with little or no variation day after day, for generally not less a period than two weeks, we may well imagine the enormous quantity of the narcotic that may be used with impunity, especially when the system is fully under the influence of an agent like the poison of typhoid fever. I have never known anything more serious than moderate dilation of the pupils (which I look upon as an admirable criterion of the safety of its therapeutic action) and slight dryness of the fauces to attend its administration.

The most remarkable effects of belladonna, perhaps, are shown by the suddenness with which the patient recovers his intellectual faculties, and the full power and control of his muscles. The latter he often takes pleasure in exercising by rapidly flexing and extending his arms—a circumstance which he adroitly wields as an argument in his appeals to the physician to allow him to get up and resume his ordinary vocation. All this was well exemplified in a stalwart Englishman, a patient of Dr. Samuel Giles, of New Cross, Deptford, whom I attended as *locum tenens* a little over a year ago.

As belladonna compleiely prevents the specific lesions in all the tissues and organs of the body naturally consequent upon the absorption of the typhic poison, it becomes an important question to decide whether the patient is rendered obnoxious or not to subsequent attacks. This is an enigma which neither experience or observation, so far, has enabled me to solve. Reasoning, however, from analogy, we may expect the disease to reappear, if at all, under a modified and milder form.

When is it safe to withhold the belladonna from the patient? As nervous symptoms resembling those following the sudden suspension of an habitual stimulant are apt to supervene upon the abrupt cessation of the drug, I deem it prudent, after the disease has continued about twenty-one days, and the eruption and other accidents have entirely disappeared, to diminish the dose, and extend the intervals of its exhibition gradually. This may be safely and conveniently done during the whole period of convalescence.

As regards the matter of stimulants, I absolutely interdict them in every form and shape to patients while under the treatment of

Belladonna, as one of my objects is to see that neither its action is masked, nor its virtues rendered doubtful, by the complexity of remedial agents. I occasionally permit a teaspoonful or so of milk punch to be given at long intervals, certainly not with a view of thereby benefiting the patient very materially, but as a *placebo* covertly addressed to the minds of over-anxious friends.

The regimen of patients, while under treatment, should be attended to with the most scrupulous care, as the slightest excess, either in eating or drinking, is sure to be followed by an attack of indigestion, and an aggravation of all the febrile symptoms. I have found the following bill of fare to answer admirably in most cases:—A quart of soup, made from cow-heel and a pound of beef; a pint of milk boiled with arrow-root; an ounce of cocoa, prepared in a pint of milk. These, together with a slice of toast, and a little salt and pepper, constitute the ordinary daily rations for an adult. The proportions must of course vary in many cases, but the articles themselves will be found well adapted to the debilitated powers of digestion commonly arising in the course of continued fever.—*Medical Times and Gazette, Feb*. 5, 1870, *p*. 146.

BRAITHWAIT'S RETROSPECT.

